# Validation of CyTOF Against Flow Cytometry for Immunological Studies and Monitoring of Human Cancer Clinical Trials

**DOI:** 10.3389/fonc.2019.00415

**Published:** 2019-05-17

**Authors:** Ramy Gadalla, Babak Noamani, Bethany L. MacLeod, Russell J. Dickson, Mengdi Guo, Wenxi Xu, Sabelo Lukhele, Heidi J. Elsaesser, Albiruni R. Abdul Razak, Naoto Hirano, Tracy L. McGaha, Ben Wang, Marcus Butler, Cynthia J. Guidos, Pam S. Ohashi, Lillian L. Siu, David G. Brooks

**Affiliations:** ^1^Tumor Immunology Program, Princess Margaret Cancer Center, University Health Network, Toronto, ON, Canada; ^2^Department of Immunology, University of Toronto, Toronto, ON, Canada; ^3^Program in Developmental and Stem Cell Biology, Hospital for Sick Children Research Institute, Toronto, ON, Canada

**Keywords:** CyTOF, flow cytometry, cancer clinical trials, immune studies, immunotherapy

## Abstract

Flow cytometry is a widely applied approach for exploratory immune profiling and biomarker discovery in cancer and other diseases. However, flow cytometry is limited by the number of parameters that can be simultaneously analyzed, severely restricting its utility. Recently, the advent of mass cytometry (CyTOF) has enabled high dimensional and unbiased examination of the immune system, allowing simultaneous interrogation of a large number of parameters. This is important for deep interrogation of immune responses and particularly when sample sizes are limited (such as in tumors). Our goal was to compare the accuracy and reproducibility of CyTOF against flow cytometry as a reliable analytic tool for human PBMC and tumor tissues for cancer clinical trials. We developed a 40+ parameter CyTOF panel and demonstrate that compared to flow cytometry, CyTOF yields analogous quantification of cell lineages in conjunction with markers of cell differentiation, function, activation, and exhaustion for use with fresh and viably frozen PBMC or tumor tissues. Further, we provide a protocol that enables reliable quantification by CyTOF down to low numbers of input human cells, an approach that is particularly important when cell numbers are limiting. Thus, we validate CyTOF as an accurate approach to perform high dimensional analysis in human tumor tissue and to utilize low cell numbers for subsequent immunologic studies and cancer clinical trials.

## Introduction

To discover immune correlates and biomarkers of disease requires global profiling of the immune system, the proteins differentially regulated by therapy and how these relate to disease outcome. Highly focused exploration may provide hypothesis-driven insight, but often the paradigm-altering discoveries come from unbiased global immune profiling. Flow cytometry (FC) has emerged as a key tool to profile multiple parameters of the immune system, including vital functional and exhaustion markers associated with the quality of the immune response (1). However, FC is limited by the number of parameters that can be analyzed at one time (generally 12 per staining panel). This means that stains must be broken up into groups with redundancy of many of the cell lineage markers in different stains. As a result, FC requires large sample sizes for coverage of diverse immune subsets. This is particularly detrimental for tumor biopsies where sample sizes are often limiting and the broad array of FC staining panels often cannot be performed. Further, when only a few markers can be analyzed in a single sample, researchers must design panels using a priori knowledge of marker expression patterns to characterize cells of interest. If unusual marker expression patterns are encountered, as is often the case in disease states, follow-up studies that require time-consuming design and optimization of new panels must be performed, assuming more patient sample is available.

Recently, the use of high-dimensional time-of-flight mass cytometry (CyTOF) to identify 40+ parameters simultaneously has emerged as a technique for broad-scale immune profiling and biomarker discovery (1–7). Because CyTOF allows far more markers to be measured in a single tube, fewer cells are required per experiment than would be needed for traditional FC, which would require multiple tubes (with different antibody panels) to cover the same number of markers. By incorporating a large number of parameters into single stains, CyTOF enables acquisition of large amounts of immunologic data from limited sample sizes to better understand biologic systems, response to therapy (8) and signatures of disease(1, 5–7, 9–14). Examples include characterization of intra- and inter-tumor leukemia heterogeneity that correlates with clinical outcomes(15) as well as dissections of T and NK cell subtypes with high resolution (16–19), antiviral T cell responses (5, 7, 18, 20), and immune cell signatures linked to recovery from surgery (21). Thus, CyTOF has enormous potential to discover disease associated immunologic changes in cancer, identify functional changes to guide subsequent therapy and ultimately predict therapeutic outcomes.

Both FC and CyTOF utilize antibodies to label targets on cells. For FC these antibodies are labeled with fluorophores that are excited by lasers to emit light subsequently detected by the flow cytometer. Due to the range of wavelengths of these light emissions, there is overlap in their emission spectra that must be mathematically compensated, thus limiting the number of fluorophores that can be used simultaneously. CyTOF uses antibodies conjugated to rare heavy metal isotopes that are not normally present in biological specimens. As opposed to fluorescence, CyTOF uses an atomic mass cytometer to detect the time-of-flight (TOF) of each metal. Each atom's TOF is determined by its mass, allowing the composition of metal atoms on each cell to be ascertained. Detection overlap among heavy metal isotopes is generally limited to <2% (22) rather than the 5–100% spectral overlap seen in conventional FC, and backgrounds are very low because cells do not naturally contain heavy metals. Thus, the detection of low-expression markers is greatly enhanced even on cell populations such as myeloid cells with high auto-fluorescence.

The goal of this study is to validate CyTOF against FC for use in immune profiling for clinical trials. Panels were designed to include major (and most minor) immune lineage defining markers in combination with a wide array of functional, activation, exhaustion, differentiation, chemotaxis, immunomodulatory, and senescence markers (Table 1). Overall, our results demonstrate that CyTOF faithfully recapitulates FC data in PBMC and tumor tissues, providing reliable staining of >35 parameters for high dimensional analyses for analysis of cancer clinical trials.

**Table 1:** CyTOF Panel.

**Mass and Tag**	**Specificity**	**Ab Clone**	**Replicates (PBMC)**	**Company**
89Y	CD45	HI30	10	Fluidigm
141Pr	CD45RA	HI100	7	Biolegend
142Nd	HLA-DR	L243	8	Biolegend
143Nd	CD57	HCD57	8	Biolegend
144Nd	CD33	WM53	8	Biolegend
145Nd	CD183 (CXCR3)	G025H7	8	Biolegend
146Nd	CD8α	RPA-T8	11	Biolegend
147Sm	CD4	RPA-T4	11	Biolegend
149Sm	Perforin	B-D48	8	Biolegend
149Sm	FoxP3	236A-E7	8	ThermoFisher
150Nd	CD103	B-Ly7	9	ThermoFisher
150Nd	Tbet	4B10	7	Biolegend
151Eu	CD39	A1	10	Biolegend
152Sm	CD11c	Bu15	8	Biolegend
153Eu	CD3	UCHT1	11	Biolegend
154Sm	IgM	MHM-88	11	Biolegend
155Gd	CD45RO	UCHL1	7	Biolegend
156Gd	CD14	M5E2	8	Biolegend
158Gd	CD27	O323	11	Biolegend
159Tb	CD19	HIB19	11	Biolegend
160Gd	CD25	M-A251	9	Biolegend
161Sy	Ki67	Ki67	8	Biolegend
162Dy	CD28	CD28.2	11	Biolegend
163Dy	CD137 (41BB)	4B4-1	8	Biolegend
164Dy	CD34	581	8	Biolegend
165Ho	CD279 (PD1)	EH12.2H7	9	Biolegend
166Er	Tim3	F38-2E2	7	Biolegend
167Er	CD95 (Fas)	DX2	11	Biolegend
9168Er	CD185 (CXCR5)	MU5UBEE	8	ThermoFisher
169Tm	TCRγδ	5A6-E9	11	ThermoFisher
170Er	CD152 (CTLA4)	14D3	7	ThermoFisher
171Yb	GranzymeB	GB11	9	Biolegend
171Yb	Helios	22F6	6	Biolegend
172Yb	CD127 (IL-7Rα)	EBioRDR5	11	ThermoFisher
173Yb	CD56	HCD56		Biolegend
174Yb	TIGIT	MBSA43	11	ThermoFisher
175Lu	CD274 (PDL1)	29E.2A3	5	Biolegend
176Yb	CD223 (Lag3)	7H2C65	7	Biolegend
191Ir	DNA1 (Cell ID)			Fluidigm
193Ir	DNA2 (Cell ID)			Fluidigm
196Pt	Cisplatin (Viability)			BioVision
209Bi	CD16	3G8	6	Fluidigm

**Figure 1:**
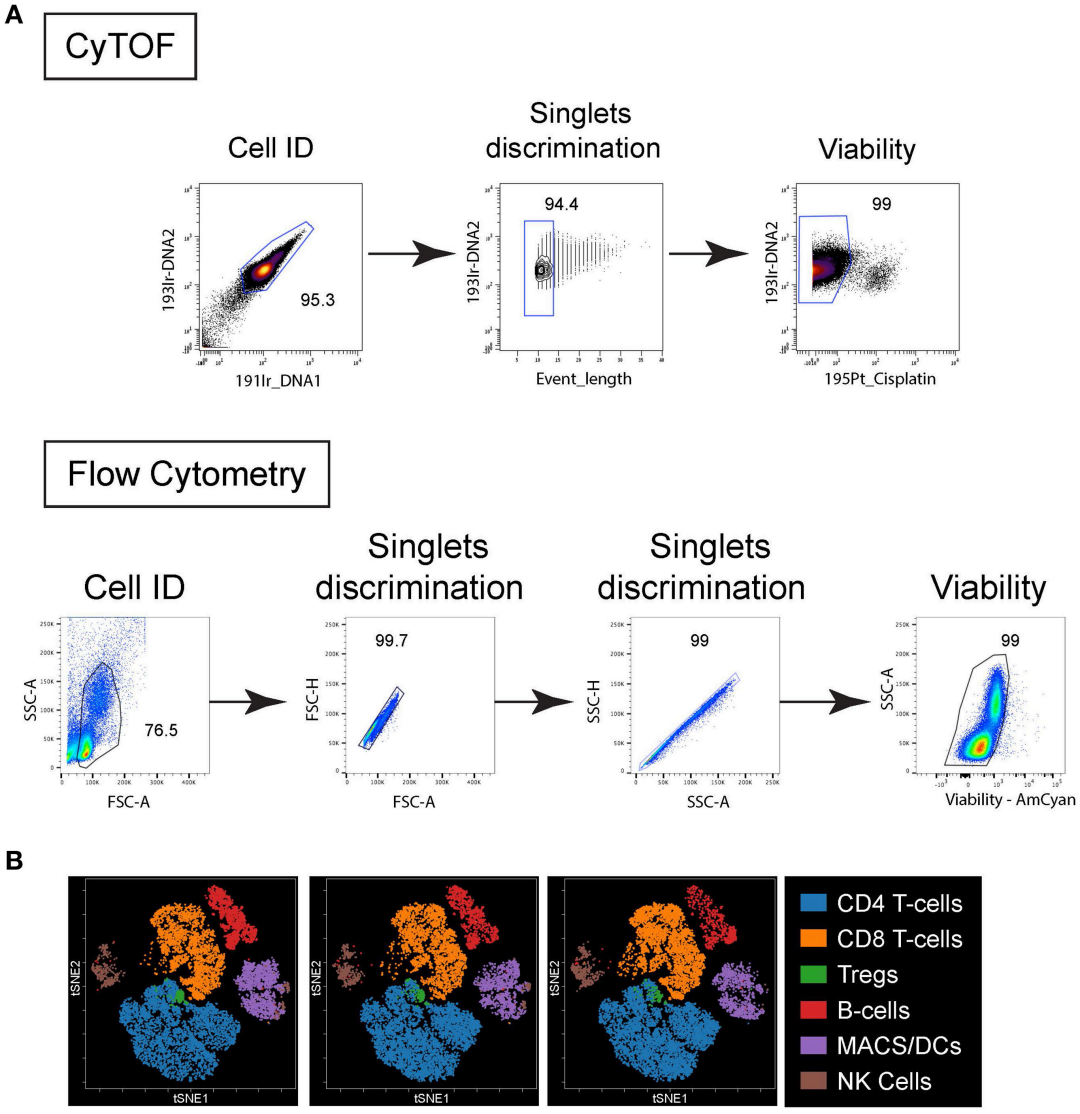
Live/Dead cell gating and population clustering. **(A)** For CyTOF (top plots), cells were first identified based on DNA staining, singlet were selected based on event length and viability based on cisplatin exclusion. For FC (bottom plots) cells were identified based on forward scatter (FSC) vs. side scatter (SSC), singlets selected based on FSC-area vs. height and SSC-area vs. height, and viability based on dye exclusion. **(B)** Immune cell populations from 4 different PBMC donors stained by CyTOF were plotted on bivariate viSNE plots. Main cell populations were manually gated based on lineage marker expression and then the manual gates were used as the overlaid (colored) dimension. The main cell populations are shown by the indicated color profile.

**Figure 2:**
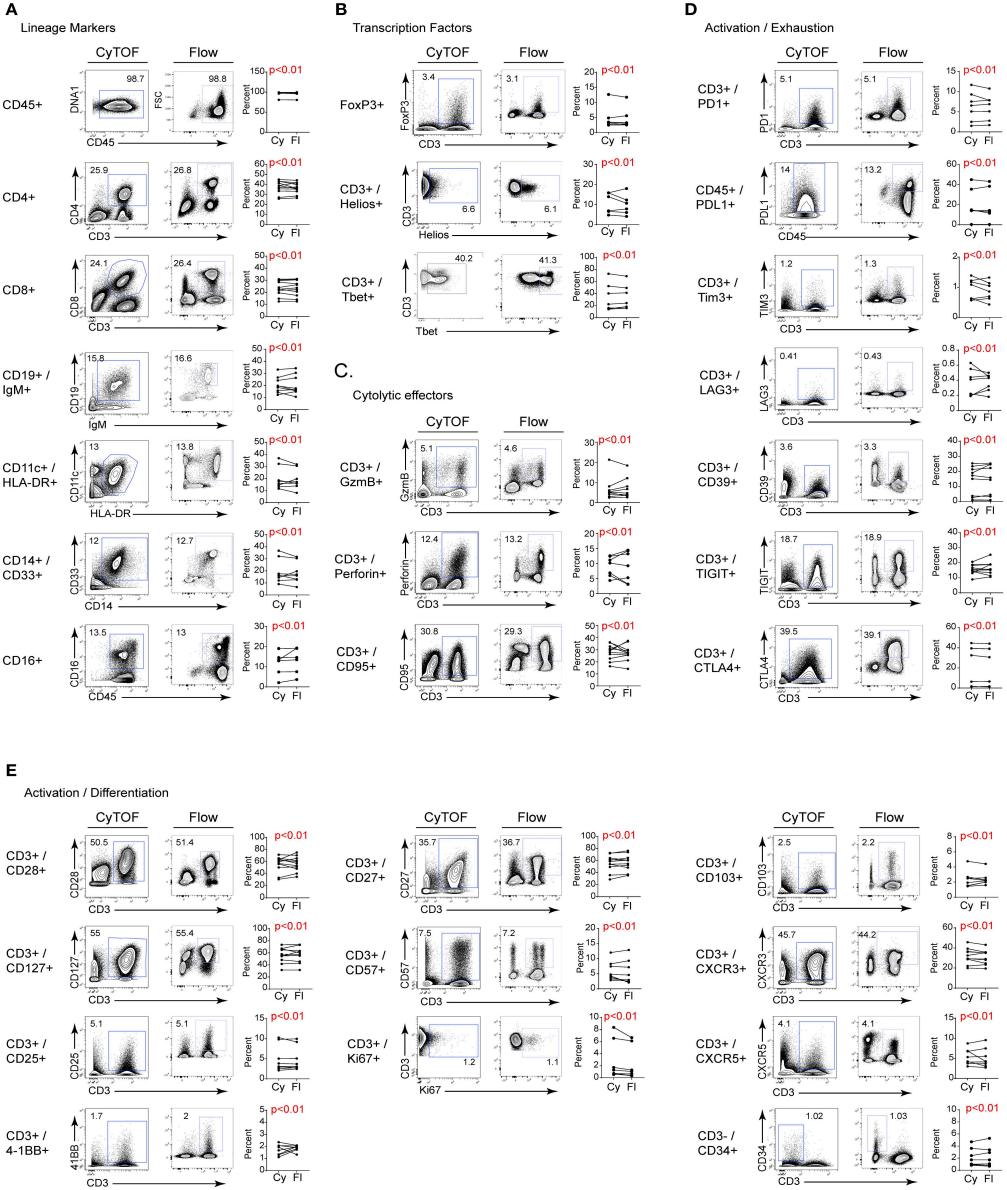
Comparison of CyTOF and flow cytometry staining of freshly isolated human PBMC. Gates in each plot show the frequency of the indicated stained protein by CyTOF (left plots) or FC (right plots) for **(A)** cell lineage defining; **(B)** transcription; **(C)** cytolytic activity; **(D)** activation/exhaustion; and **(E)** activation/cellular differentiation. Graphs display donor sample paired expression of the frequency staining positive for the indicated protein by CyTOF (Cy) and FC (Fl). The number of donors for each stain is indicated in Table 1. Significance was determined by the TOST test for equivalence. *p* ≤ 0.05 was considered statistically equivalent.

*Antibodies used and their metal conjugation are shown in the table. Replicates indicates the number of different healthy PBMC donors that were used for the validation in Figures 1, 2. Company indicates where each antibody was purchased*.

## Materials and Methods

### PBMC and Tumor Tissue Collection

All human tissues and blood were obtained through protocols approved by the institutional review board. Written informed consent was obtained from all donors. Peripheral blood samples were collected from 11 healthy donors into sterile anticoagulant-coated tubes from the Healthy Donor Blood Collection Study at the Princess Margret Cancer Center (IRB#11-0343). Five surgically resected tumor specimens; 2 ovarian (IRB#10-0335), 2 melanoma (IRB#05-0495), and 1 breast tumor (IRB#06-0801) were obtained from the UHN Biospecimen Program.

### Sample Processing

Peripheral blood mononuclear cells (PBMCs) were isolated by Ficoll-paque density gradient centrifugation from the healthy donor's blood. After isolation, cells were directly stained for flow and mass cytometry. Excess cells were aliquoted in 10^7^ cells per vial in freezing media (10% DMSO in heat-inactivated FBS) and cryopreserved in liquid nitrogen. Tissue samples were minced into 2–4 mm^3^ fragments and digested enzymatically into single cell suspensions with the gentleMACS Dissociator (Miltenyi Biotech, catalog #130-093-235) and the human tumor dissociation kit (Miltenyi Biotech, catalog #130-095-929) to obtain single cell preparations. Cells were then aliquoted and cryopreserved in liquid nitrogen.

### CyTOF and Flow Cytometry Antibodies

The same antibody clones were used for CyTOF and FC. The vendor from which each antibody was purchased is listed in Tables 1, 2. For CyTOF, purified unconjugated antibodies used were Biolegend MaxPar Ready antibodies or custom-made with no additional protein carrier from Biolegend or Thermo Fisher. CyTOF antibodies were labeled with metal-tag at the SickKids-UHN Flow and Mass Cytometry Facility using the MaxPar Antibody Labeling kit from Fluidigm (catalog #201300).

**Table 2:** Flow Cytometry Panels.

**Fluorochrome**	**Specificity**	**Company**
**FLOW CYTOMETRY PANEL 1**		
BUV395	CD3	BD Bioscience
BV605	CD4	BD Bioscience
FITC	TCRgd	ThermoFisher
PerCP	CD8	Biolegend
PE-Cy7	TIGIT	ThermoFisher
APC-Cy7	CD56	Biolegend
eFluor506	Viability	ThermoFisher
**FLOW CYTOMETRY PANEL 2**		
FITC	CD16	Biolegend
PE	CD14	Biolegend
PE-Cy7	CD11C	Biolegend
APC	PDL1	Biolegend
AlexaFluor 700	CD33	ThermoFisher
BV421	CD45RO	Biolegend
BV605	CD45	Biolegend
BV650	CD45RA	Biolegend
BV711	HLA-DR	Biolegend
eFluor506	Viability	ThermoFisher
**FLOW CYTOMETRY PANEL 3**		
BUV395	CD3	BD Bioscience
BV421	CD127	ThermoFisher
BV605	CD4	BD Bioscience
AlexaFluor700	CD8	ThermoFisher
PerCP-eF710	CD39	ThermoFisher
PE-CF594	CD95 (Fas)	BD Bioscience
eFluor506	Viability	ThermoFisher
**FLOW CYTOMETRY PANEL 4**		
BUV395	CD3	Biolegend
PE	CD103	ThermoFisher
PE-Cy7	CD28	ThermoFisher
APC	CXCR5	ThermoFisher
PerCP	CD8	Biolegend
AlexaFluor700	CD4	ThermoFisher
BV605	CXCR3	Biolegend
EFluor506	Viability	ThermoFisher
**FLOW CYTOMETRY PANEL 5**		
BUV395	CD3	BD Bioscience
BV605	CD4	BD Bioscience
PE	CD57	Biolegend
PerCP	CD8	Biolegend
APC-Cy7	CD27	Biolegend
BV421	IgM	Biolegend
AlexaFluor400	CD34	Biolegend
AlexaFluor700	CD19	ThermoFisher
eFluor506	Viability	ThermoFisher
**FLOW CYTOMETRY PANEL 6**		
BUV395	CD3	BD Bioscience
BV605	CD4	BD Bioscience
PE	FoxP3	ThermoFisher
PerCP	CD8	Biolegend
APC	CD25	Biolegend
APC-eF780	Helios	ThermoFisher
BV421	Tbet	Biolegend
eFluor506	Viability	ThermoFisher
**FLOW CYTOMETRY PANEL 7**		
BUV395	CD3	BD Bioscience
BV605	CD4	BD Bioscience
FITC	Perforin	Biolegend
PE	GranzymeB	ThermoFisher
PerCP	CD8	Biolegend
eFluor506	Viability	ThermoFisher
**FLOW CYTOMETRY PANEL 8**		
BUV395	CD3	BD Bioscience
BV421	Tim3	Biolegend
BV605	PD1	Biolegend
BV711	Ki67	Biolegend
PE	CD137	ThermoFisher
PerCP	CD8	Biolegend Biolegend
AlexaFluor700	CD4	ThermoFisher
PE-Cy7	Lag3	Biolegend
EFluor660	CTLA4	ThermoFisher
eFluor506	Viability	ThermoFisher

*Eight different panels were used to span the antibodies needed for the single CyTOF panel. Each panel indicates the antibodies and their fluorescent conjugation*.

### Staining Procedure

After PBMCs isolation, cells were counted and viability measured by trypan blue exclusion. One million viable cells were aliquoted into 4 ml polystyrene V-bottom tubes for CyTOF staining. For FC staining, 1 million viable cells per well were added to 8 wells of into 96-well plate for the FC panels shown in Table 2. CyTOF and FC staining were performed simultaneously. Single cells suspensions from tumor tissues were handled analogously same way after thawing.

For FC staining, cells were incubated in Fc blocker (ThermoFisher, catalog #16-9161-73) for 10 min at room temperature, followed by incubation in the surface markers antibody cocktail for 30 min at 4°C. Cells were then fixed with 4% paraformaldehyde (PFA). For intracellular staining, cells were fixed and permeabilized by incubation with eBioscience Foxp3/Transcription Factor Staining Buffer Set (catalog #5523) for 30 min at 4°C, followed by incubation with intracellular antibody cocktail. Cells were washed by centrifugation at 480 × g for 3 min in phosphate buffer saline (PBS) to be ready for FC acquisition.

For CyTOF staining, cells were Fc blocked as for FC staining, followed by incubation with surface marker staining cocktail for 30 min at 4°C. For viability staining, cells were washed with PBS and incubated for 5 min in room temperature in 200 μl of 1 μM cisplatin solution (BioVision, catalog #1550-1000). Cisplatin was quenched by adding 2 ml of 5% serum-containing PBS. Cells were fixed and permeabilized immediately in eBioscience Foxp3/Transcription Factor Staining Buffer Set, followed by incubation in the intracellular markers antibody cocktail for 30 min at 4°C. EQ Four Element Calibration Beads (Fluidigm) were used to normalize signal intensity over time. For iridium labeling of cellular DNA, cells were suspended in 1 ml of 100 nM of iridium (Fluidigm, Catalog #201192B) in PBS containing 0.3% saponin and 1.6% formaldehyde for 1 h at 4 °C. Cells were then washed and kept in PBS with 1.6% formaldehyde in 4°C for 1 to 4 days before acquisition.

### Data Acquisition and Analysis

Cells stained for FC were acquired on the day of or the day after staining using a 5-laser LSR Fortessa X-20 (BD) at the Flow Cytometry Core Facility at Princess Margaret Cancer Center. Single stain controls for each fluorochrome were prepared using UltraComp eBead Compensation Beads (ThermoFisher, catalog #01-2222-42). Data were analyzed using FlowJo V10.

For CyTOF data acquisition, cells were pelleted in Milli-Q water on the day of acquisition and transferred on ice to SickKids-UHN Flow and Mass cytometry Facility to be acquired on third-generation Helios mass cytometer (Fluidigm). Cells were then resuspended into 1 ml of EQ beads diluted 1:10 in Maxpar Cell Acquisition Solution and filtered through cell strainer cap tubes. Cells were acquired at rate of 100–250 events per second. Acquired raw FCS files were normalized with the preloaded normalizer algorithm on CyTOF software version 6.7. Normalized CyTOF FCS files were analyzed using Cytobank 6.2 (Cytobank, Inc) to manually gate different populations and create 2 dimensions and high dimensional plots. Parameters used for making the viSNE plots are CD3, CD4, CD8, CD25, Foxp3, CD19, CD56, CD16, HLA-DR, CD11c, CD33, CD14. Populations were then defined based on known lineage combinations of these proteins. viSNE analyses were performed using equal sampling per comparison, perplexity = 30, theta = 0.5, iterations = 1,000–5,000. Figure 1 viSNE: event sampling = 49,998 cells per sample, 5,000 iterations, final KL divergence: 2.42. **Figure 4** viSNE: event sampling = 30,664 cells per sample, 2,000 iterations, final KL divergence: 2.53. **Figure 5** viSNE: event sampling = 6,910 cells per sample, 1,000 iterations, final KL divergence: 1.26.

For both FC and CyTOF, 1 million cells were stained and an average of 100,000 cells were acquired. Populations were gated based on their expression of linage defining markers (e.g., CD3 for T cells, CD19 for B cells) For manual gating on biaxial plots, the positive population of each marker (e.g., CD3+GzmB+) was defined as the events above the negative population (e.g., CD3-) on the same plot for both CyTOF and FC.

Raw flow cytometry and CyTOF data files from all experiments described herein are publicly available at www.flowrepository.org; http://flowrepository.org/id/RvFrkC3p2UldoMbc7kQpqboaZ6UYvg4alJi8JFKywZUnNbhULFhcOoSLOeDJVtwf.

### Statistics

The equivalency between CyTOF and FC were compared using a paired TOST equivalence test. The paired TOST equivalence test reverses the null and alternative hypothesis to place the burden of proof on showing that two variables measured for the same subject are significantly equivalent (23). R package “equivalence” (version 0.7.2) was used to perform the equivalence test (24). We used an epsilon value of 5, indicating that a difference in proportion smaller than 5% is deemed equivalent. *P*-value ≤ 0.05 was considered statistically equivalent. GraphPad Prism 6 software (GraphPad Software, Inc.) was used to perform Pearson's correlation test.

## Results

### Comparison of CyTOF vs. Flow Cytometry Staining in Freshly Isolated Peripheral Blood Mononuclear Cells

To appropriately compare staining patterns and expression of proteins of interest for cancer immunotherapy trials, we developed a 40+ parameter CyTOF panel that could identify all major (and most minor) cell lineage defining markers, in combination with transcription factors, activation/exhaustion, differentiation, and cytolytic factors (Tables 1, 2). These markers were chosen to broadly profile the differentiation and functional state of many cell types simultaneously instead of solely focusing on a single cell type (such as CD8 T cells or macrophages) as is often the case. For comparison of CyTOF to FC, the same antibody clones were used. Titrations were separately performed for CyTOF and flow cytometry antibodies to obtain the optimal concentration for use. In general, similar concentrations were optimal for both assays.

Comparisons were first performed using peripheral blood mononuclear cells (PBMC) isolated from healthy individuals. The PBMC were obtained, isolated and stained by flow cytometry or CyTOF on the same day. Standard FC utilizes the forward light scatter (cell size) and side light scatter (cells internal complexity/ granularity) to identify intact cells and from debris (Figure 1A). These same parameters are not feasible using mass cytometry, so a DNA-intercalator containing two iridium isotopes (191Ir and 193Ir) is used to detect cells by the CyTOF instrument (Figure 1A). These reagents additionally can be used for comparison with event length to distinguish single cells, doublets and other non-cellular particles (Figure 1A). Fluorescent reagents that are preferentially taken up by dead cells are used to distinguish live from dead cells by FC (Figure 1A). Similarly, short treatment of cells with the platinum-based reagent cisplatin is used in CyTOF to distinguish live from dead cells (25) (Figure 1A). For CyTOF, metal-containing beads (EQ Calibration beads from Fluidigm) are added to each sample to normalize signal variation (i.e., intensity of signal detected in each metal isotope “channel”) resulting from instrument variability over time within each acquisition and between different samples acquired on the same day. In CyTOF, crosstalk between different mass channels can occur mainly due to potential isotopic impurities in the channels that detect other isotopes of the same element. Also, in cases of extremely high signal intensity, spillover, mainly in the mass (M) +1 and M-1 channels can occur as the instrument detectors becomes unable to separate ion peaks of adjacent channels. Another source of spillover in the M+16 channel occurs due to variable oxide formation (13). At the beginning of each analysis, any spillover was determined for each M+1, −1 and +16 channel and if observed, that channel was not used for subsequent analysis in the stain. Note, spillover was not observed in the experiments using this panel. Dimensionality reduction of the CyTOF data onto t-distributed stochastic neighbor embedding (t-SNE)–based visualization (viSNE) maps were used to simultaneously resolve the many distinct immune populations (Figure 1B) in combination with the numerous phenotypic/functional markers included in the panel, something less feasible by FC due to the restrictions in parameters that can be easily included in a given stain.

To compare the staining of individual proteins by FC and CyTOF, we directly measured their expression using bivariate dot plots. As shown in Figure 2, the frequency of cells expressing a given protein statistically equivalent between CyTOF and FC. We determined statistical equivalence by using the TOST equivalence test, which returns *p*- values below the significance threshold if the two proportions are deemed equivalent. This similarity was true whether the protein of interest was expressed on the cell surface or intracellularly (Figure 2 and Figure S1). Further, a similar staining frequency of positive staining cells was observed whether the marker was expressed at high (e.g., CD28, CD127) or low (e.g., CD25, 4-1BB) levels (Figure 2 and Figure S1). Visually, a few bivariate plots do not show the exact same staining pattern/intensity between CyTOF and FC, even though frequencies are equivalent (e.g., Helios, T-bet).

We next measured the change in staining intensity of each marker by flow cytometry and CyTOF comparing the mean fluorescence intensity (MFI) and the mean metal intensity (MMI), respectively, of each protein. This was done by gating on the negative and positive staining populations for each sample using the same logarithmic scale (same high and low end) for FC and CyTOF data, and then calculating the fold change. This approach was used instead of simply stating the MFI/MMI of the positive population to account for differences in the non-specific antibody binding, the background (autofluorescence or metal content) or due to inherent differences in the “brightness” of a given fluorochrome or metal tag. The fold change of a given protein was either the same between CyTOF and FC, or was higher by CyTOF (Table 3). It should be noted however that CyTOF background medians are often zero or close to zero, thereby increasing the fold change values for the CyTOF data. Thus, for the staining of human PBMC for cell lineage, activation, exhaustion, differentiation, and functional proteins of interest for immune monitoring and discovery in cancer immunotherapy trials, CyTOF data provides the same quality of staining as flow cytometry. Further, the ability to combine all the markers into one stain using CyTOF provides the opportunity to simultaneously measure changes across the immune system and to identify changes without preconceived bias of what proteins a cell “should” or “should not” express.

**Table 3:** Comparison of MFI and MMI from healthy PBMC donors.

	**Population**	**Fold change MFI+/− SE**	**Fold change MMI+/− SE**
LAG3	CD3+ T-cells	54.3 ± 11.7	58.5 ± 12.5
CD27	CD3+ T-cells	72.3 ± 14.8	87.2 ± 12.2
CD28	CD3+ T-cells	63.7 ± 12.5	63.2 ± 8.3
CD25	CD3+ T-cells	26.76 ± 6.4	168 ± 73.4
4-1BB (CD137)	CD3+ T-cells	20.9 ± 3.8	167.8 ± 48.2
TCRγδ	CD3+ T-cells	38.8 ± 3.6	489.6 ± 97.6
CD57	CD3+ T-cells	167 ± 52.7	177.1 ± 36.5
CD103	CD3+ T-cells	186.8 ± 32.5	148.1 ± 43.4
Ki67	CD3+ T-cells	86.3 ± 70.2	38.9 ± 13.2
PD-1	CD3+ T-cells	9.3 ± 2.8	33.8 ± 6.1
CTLA4	CD3+ T-cells	10.9 ± 2.6	18.8 ± 5.6
CD127	CD3+ T-cells	7.7± 1.0	56.3 ± 12.3
TIGIT	CD3+ T-cells	55.0 ± 4.4	32.7 ± 5.4
TIM3	CD3+ T-cells	10.3 ± 1.5	167.9 ± 28.9
CD39	CD3+ T-cells	35.7± 11.9	29.6 ± 7.3
FOXP3	CD3+ T-cells	6.2 ± 0.7	41.0 ± 13.3
Granzyme B	CD3+ T-cells	52.9 ± 9.3	45.5 ± 6.2
Perforin	CD3+ T-cells	6.2 ± 0.9	21.6 ± 9.2
Fas	CD3+ T-cells	92.5 ± 33.4	72.2 ± 16.2
T-bet	CD3+ T-cells	4.8 ± 0.5	6.7 ± 1.4
Helios	CD3+ T-cells	12.3 ± 1.7	69.3 ± 42.7
CXCR5	CD3+ T-cells	37.2 ± 11.3	72.0 ± 19.1
CXCR3	CD3+ T-cells	11.7 ± 1.2	93.5 ± 25.1
PDL1	CD45+	83.5 ± 32.2	123.4 ± 24.7
CD45RA	CD45+	46.9 ± 17.2	80.0 ± 23.17
CD45RO	CD45+	56.6 ± 13.3	54.3 ± 11.6
IgM	B-cells	131.3 ± 14.9	199.8 ± 40.8

*The Mean Fluorescence Intensity (MFI; flow cytometry) and the Mean Metal Intensity (MMI; CyTOF) were calculated for each stain. This was done by gating on the positive and negative populations for each stain and then calculating the fold change of positive staining over negative staining. The fold change shows the staining intensity of each antibody used, and that fluorescent and metal-tagged antibodies perform similarly in most cases, if not better by some CyTOF antibodies*.

### Comparison of CyTOF and Flow Cytometry in Frozen PBMC

**Figure 3:**
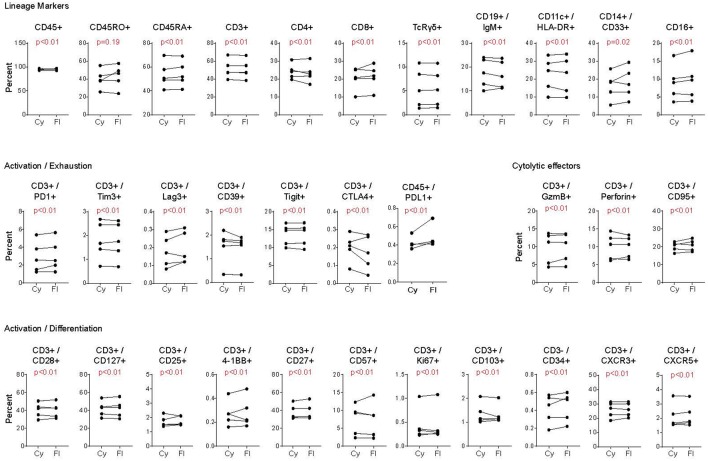
Comparison of CyTOF and flow cytometry staining of viably frozen human PBMC. Analysis was performed as in Figure 2, except using PBMC that had been previously viably frozen. Each graph represents the donor paired frequency of cells staining positive for the indicated marked by CyTOF and FC. Data represent previously frozen PBMC samples from 5 healthy donors. Significance was determined by the TOST test for equivalence. *p* ≤ 0.05 was considered statistically equivalent.

Freezing of cells can lead to changes in protein detectability, however these are generally due to cleaving or loss of surface expression as opposed to changes in the technical aspects of the assay (26–28). As a result, we next compared whether CyTOF and FC were similarly effective using previously frozen PBMC. Note, the goal of this comparison is not to determine if freezing of cells disrupts certain markers, but instead to determine whether the two cytometric techniques perform equivalently on previously frozen cells. Viably frozen PBMC from healthy donors were thawed and stained for FC and CyTOF. For these analyses, PBMC had been frozen for at least 1 month prior to thawing and staining. Analogous to fresh PBMC, the percentage of positive cell staining for each marker was similar by CyTOF and FC, despite the inter-individual variability for each marker (Figure 3). Further, similar to fresh PBMC, the staining intensity (i.e., the fold change in MFI and MMI) was similar or better using CyTOF (not shown). Thus, CyTOF is a robust approach to quantify cellular presence, phenotype and function in previously frozen PBMC.

### Titration of PBMC Required for CyTOF Analysis

**Figure 4:**
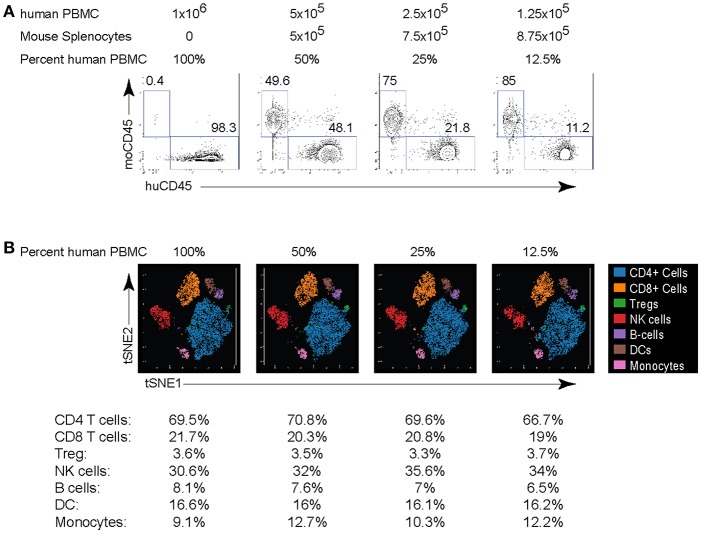
Titration of PBMC for CyTOF staining. Human PBMC isolated from healthy donors were serially 2-fold diluted in mouse splenocytes at the indicated ration to maintain a total cell count of 1 million cells per stain. **(A)** Human and mouse cells were differentiated based on their expression of CD45. Numbers in the plots indicate the frequency of each population in the indicated dilution. **(B)** viSNE plots of major human PBMC populations from each dilution. The numbers under the viSNE plots indicate the frequency of each population in the dilution and the numbers in parenthesis indicate the number of cell events in the subset. Data are representative of 2 experiments using human PBMC from different donors.

A critical issue limiting studies with small numbers of cells is the increased cell loss with staining procedures, the potential for increased “background” staining and for CyTOF in particular, the higher cell loss during acquisition. To overcome this issue, we developed a strategy in which serially diluted numbers of human PBMC were mixed with mouse splenocytes at a ratio such that the final number was always a million cells. Mouse splenocytes were used because they can be reliably distinguished from human hematopoietic cells based on expression of non-cross reactive clones of anti-mouse CD45 and anti-human CD45 antibodies (Figure 4A). We performed two-fold dilutions of human PBMC resulting in human cells comprising 100% (1 million PBMC), 50%, 25% or 12.5% (125,000 PBMC) of the total cells in the mix. These dilutions were subsequently stained for CyTOF analysis using the panel in Table 1 and acquired. Comparison of the anti-mouse and anti-human CD45 antibody expression demonstrated the expected ratios of human PBMC based on the starting dilution and this was observed even at the lowest dilution containing only 125,000 human PBMC (Figure 4A). Further, titration down to 125,000 human PBMC did not alter their proportions or lead to loss of the smaller populations (Figure 4B). Of course, biologic restrictions still apply and at diminishing numbers of cells, small populations will increasingly fall below detection due to their loss in the population, similar to FC. Thus, by adjusting the cell numbers to maintain 1 million cells per stain, reliable CyTOF data can be obtained from as few as 125,000 human PBMC. This technique will be helpful in situations where cell numbers are limiting due to biologic restrictions (e.g., tumor biopsies) or multiple analyses are desired from a limited number of cells.

### Validating CyTOF in Tumor Tissues

**Figure 5:**
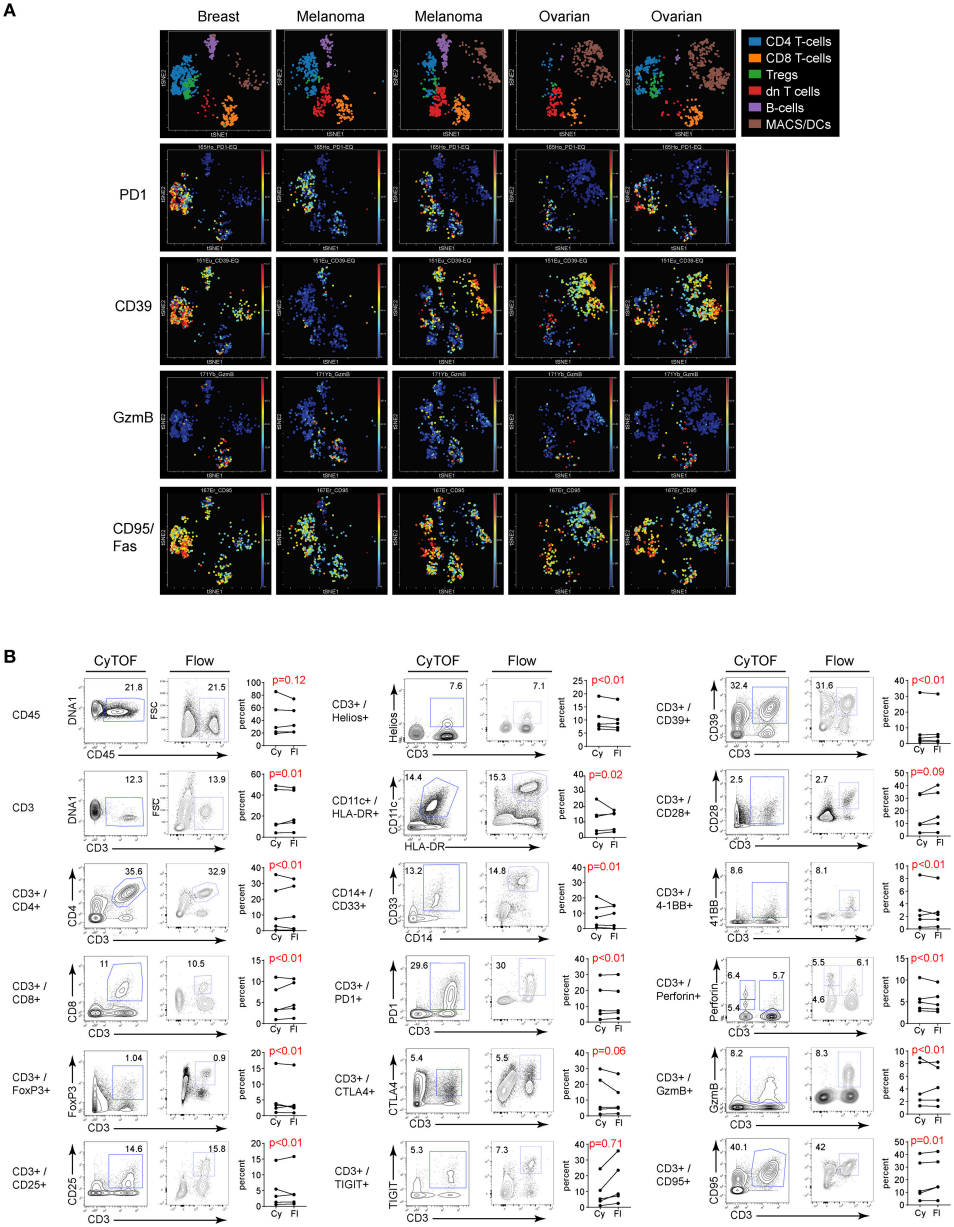
Flow vs. CyTOF staining of tumor infiltrating leukocytes. Single cell suspensions of previously viably frozen tumors were stained for CyTOF and FC. Five tumors were chosen for analysis: 2 melanoma, 2 ovarian, and 1 breast. **(A)** CyTOF viSNE plots of major immune cell populations and expression of selected proteins from the tumor tissues gated on CD45 positive cells. dn T cells: CD3+, but CD4 and CD8 double negative T cells. **(B)** Gates in each plot show the frequency of the indicated stained protein by CyTOF (left plots) or FC (right plots). Graphs display donor sample paired expression of the frequency staining positive for the indicated protein by CyTOF (Cy) and FC (Fl). Significance was determined by the TOST test for equivalence. *p* ≤ 0.05 was considered statistically equivalent.

Many studies have used CyTOF to interrogate tumor tissues, yet a direct comparison of its validity compared to FC in human tumor samples is lacking. To validate CyTOF vs. FC in human tumor tissues, we used single cell suspensions of previously viably frozen tumors. For our analysis, we chose to compare five tumors made up of 3 types: 2 melanoma, 2 ovarian and 1 breast tumor. Initial viSNE analysis showed various amounts of inter-patient variability, but in all cases major immune cell populations were resolved, including T cells, Tregs, macrophages and MDSC (Figure 5A). Further, within these various populations, phenotypic, functional, and activation/exhaustion proteins with broad or restricted distribution could be identified, including high expression of PD1 in tumor infiltrating CD4 T cells and Tregs, with less PD1 expression observed in CD8 T cells, high level, and broad CD95 (Fas) and CD39 expression across many populations of tumor-infiltrating cells (although the latter was largely absent from CD8 T cells), and restricted expression of granzyme B, primarily by CD8 T cells (Figure 5A).

Direct evaluation of CyTOF and FC staining in the different tumor types demonstrated similar proportions of immune cells in the tumor based on CD45 expression. Within the immune cell populations, staining for individual cell subsets was comparable between the two techniques (Figure 5B and Figure S2). Importantly, comparably to flow cytometry, CyTOF identified expression of numerous activation, differentiation, and functional proteins on tumor infiltrating cells (Figure 5B and Figure S2), and did so in a single stain as opposed to FC which required many separate panels with duplicate lineage markers to attain this same level of staining (Tables 1, 2). Further, like PBMC, the fold change in MFI and MMI were similar or elevated with the CyTOF stain in the tumors (not shown).

Our results show that the two technologies provide highly equivalent values across markers and populations in fresh and frozen PBMC, and tumor biopsies. Values of few populations (CD45RO+ in Frozen PBMC, and CD45+, CD3+TIGIT+, CD3+CTLA4+, and CD3+CD28+ in tumor biopsies) did not give rise to statistically equivalent results using equivalence test. However, the values of these populations from CyTOF and FC showed highly significant correlation using Pearson correlation test as shown in Table 4 (*r* ranges from 0.92 to 0.99, *p* < 0.05). We believe that the statistical inequivalence we observe in these populations is not due to differences in the two technologies. Instead, the sample size (*n* = 5) for both the frozen and biopsies specimens did not allow the values of these populations to reach the level of statistical significance using the TOST test for equivalence, although they correlated with high significance using the Pearson test and the fresh samples (*n* = 11) showed highly significant values for all populations.

**Table 4:** Statistical correlation between some CyTOF and Flow populations.

	**Sample type**	**Pearson correlation**	
		***r***	***p***
CD45RO+	Frozen PBMC	0.92	0.02
CD45+	Biopsy	0.99	0.000
CD3+ TIGIT+	Biopsy	0.97	0.006
CD3+ CTLA4+	Biopsy	0.97	0.005
CD3+ CD28+	Biopsy	0.98	0.002

*Pearson correlation coefficient was calculated for populations that did not reach statistical significance using the TOST test for equivalence to indicate how closely their values are correlated*.

## Discussion

The ability of CyTOF to combine many parameters into a single panel allows an unbiased and efficient approach for discovery of novel disease-associated cell populations or biomarkers from limited tumor samples (29). Yet, the comparability of CyTOF to the more standard use of FC of these tumor studies has not been stringently validated. Herein, we demonstrate that using our 40+ parameter panel on PBMC and tumor tissue samples, CyTOF is at least as effective, if not more so, than FC for the identification of diverse cell subsets and their subsequent phenotyping. To validate the use of CyTOF we developed a 40+ parameter panel analyzing diverse cell lineages in combination with a comprehensive panel of differentiation, transcription, chemotactic, activation, exhaustion, senescence and functional factors, chosen for their observed and potential relevance for monitoring and discovery in cancer clinical trials. Both CyTOF and FC had comparable efficacy to identify proportions of cell subsets in human PBMC and tumors, including multiple subsets critical to cancer control and the immunotherapeutic response; e.g., T cells, Tregs, dendritic cells, macrophages, and MDSCs. These techniques were equally efficient whether the PBMC were fresh or previously viably frozen. On these subsets, proteins associated with cell function and differentiation state were stained with the same or better fidelity by CyTOF compared to flow cytometry. Since small numbers of cells are often obtained from tumor tissues, it is important to note that reliable data could be observed by CyTOF using as few as 125,000 PBMC when they were pre-mixed with carrier mouse splenocytes prior to staining to increase overall cell numbers and minimize the loss of human cells during staining procedures and washing steps. In addition, non-immune cells (including non-hematopoietic derived tumor cells) can also be identified based on the lack of CD45 expression or by addition of other tumor-antigen specific antibodies. Importantly, CyTOF was able to simultaneously measure all these parameters whereas FC required multiple panels with significant overlap to achieve this goal. This allowed detailed high-dimensional analyses to be performed and a large number of immune cell populations to be plotted on bivariate viSNE plots for subsequent interrogation. This approach is beneficial for immune monitoring, mechanistic understanding and biomarker discovery because it provides an unbiased and broad analysis of the immune system with combinations of markers that do not rely on a priori decisions of cell attributes.

Currently, most of the isotopic metals commercially available for conjugation with antibodies are from the lanthanides series. A panel of 40 antibodies can be used simultaneously without technical difficulties; alongside DNA parameters to identify cells, and viability dye to distinguish live from dead cells. Research is underway in the polymer chemistry field to develop use of metals from outside the lanthanides series, to increase the number of parameters researchers can use per panel. The cost of metal tagged-antibodies, antibodies conjugation kits, and the running reagents are quite high and may be impeding the widespread of CyTOF use. Hopefully, with increasing demands, and advancement in reagents and instruments manufacturing technology, prices will be more affordable to wide range of laboratories.

Designing an optimal CyTOF panel is as important as it is in flow cytometry. Although technically there is no signal interference between mass channels, isotopic impurities can cause a small amount of contamination between different channels. Therefore, the isotopic purity of the metal-tags used must be taken into consideration when assigning cellular biomarkers to each metal. Generally, less pure metals should be paired with low expression biomarkers, as this keeps the spillover at the background level in the channels where spillover is anticipated and reduces signal interference. Similar to flow cytometry, markers with low expression ideally are paired with high signal-intensity metals like 165Ho or 169Tm for better gating and resolution of the positive population. Again, like flow cytometry, to reduce signal “spillover” in CyTOF, it is good practice to try to use of markers exclusive to cell populations (e.g., CD3, CD19) on adjacent channels where “spillover” potential is highest. Antibody titration to find the optimal dilution is also equally important, as lower dilutions will result in lower resolution, while higher dilution will increase background and “spillover” on these susceptible channels.

The photobleaching process of fluorescent dyes in flow cytometry makes it paramount to acquire samples within a few hours after staining. On other hand, metal-tagged samples can be run up to 2 weeks after staining without notable loss of signal and can be cryopreserved up to 1 month without affecting the data quality or staining integrity of both surface and intracellular markers (30). This is very useful in clinical trials, wherein long-term preservation allows researchers to collect samples over a period of time and acquire them simultaneously.

The data analysis of CyTOF is perhaps the most challenging part of the workflow. With cytometry data in general, manual gating is the one of the main contributor to inter-laboratory variations (31). An optimally designed panel, with a well-matched biomarkers and metals-tags as mentioned above, will cause less trouble gating and resolving positive events. So, efforts must be made to design an optimal panel for good data quality. Some laboratories use mass minus one controls (similar to fluorescence-minus one in FC) to build a hierarchy of gates and set positivity threshold, but this does not take into account the inherent background staining of each antibody and the non-specific binding (even if isotype-matched control antibody is used) which leads to significant false positive signal. Further, it is impractical to prepare mass-minus one control for 40+ antibodies. However, mass-minus one controlling is ideal to investigate a potential spillover between channels. Fortunately, the unsupervised clustering and automated populations-detection algorithms, which accompanied the advent of CyTOF high-dimensional data, have decreased the need for manual gating (32). However, for the purpose of this article, we test the similarity between CyTOF and flow cytometry on a marker-by-marker basis and representative examples of the gating used for each marker are shown and scatter plots provided. Thus, manual gating on biaxial plots was employed with experience and knowledge of the brightness and purity of each of the metal tags used, their intrinsic background, and crosstalk, and also, familiarity with the staining pattern of each immune marker and the frequencies of cell populations.

A major drawback to CyTOF is acquisition flow rate. In comparison to flow cytometry (thousands of cells/second), CyTOF has a slower flow rate (~250–500 cells/second), resulting in longer acquisition time. Additionally, sample preparation for CyTOF requires extra caution to avoid contamination with heavy metals, which are common ingredient in laboratory detergents and other basic reagents. Further, because cells are ultimately vaporized in CyTOF, sorting out populations of interest for further analysis and downstream applications is not possible. This is something flow cytometers are able to do easily. Finally, the increase in number of parameters, made available by CyTOF technology, has intensified the complexity of data, which is a strong attribute of the technique, but also requires deeper analysis and in many cases new bioinformatics approaches to interpret and visualize the data. Importantly, neither FC nor CyTOF is the superior technique for all applications; rather, the choice must rest on the questions asked, the answers sought and the ability to analyze different data sets in meaningful ways. Thus, our data now validate CyTOF as an accurate approach to perform high dimensional analysis in human PBMC and tumor tissue for immunologic studies and cancer clinical trials.

## Ethics Statement

All human tissues and blood were obtained through protocols approved by the Princess Margaret Cancer Center/University Health Network institutional review board. Written informed consent was obtained from all donors. All studies were performed using de-identified human data.

## Author Contributions

RG, BN, LS, and DB designed research. RG and HE performed experiments. RG, BN, and DB analyzed data. BM, RD, MG, WX, SL, HE, AR, NH, TM, BW, MB, CG, PO, LS, and DB provided intellectual input, critical discussion, and contributed technical expertise and discussion. RG and DB wrote the paper.

### Conflict of Interest Statement

The authors declare that the research was conducted in the absence of any commercial or financial relationships that could be construed as a potential conflict of interest.

## Supplementary Material

Figure S1 Comparison of CyTOF and flow cytometry staining of freshly isolated human PBMC- Additional proteins. Related to Figure 2. Gates in each plot show the frequency of the indicated stained protein by CyTOF (left plots) or FC (right plots). Stains were performed in the same set as shown in Figure 2. Significance was determined by the TOST test for equivalence. *p* ≤ 0.05 was considered statistically equivalent.

Figure S2 Comparison of CyTOF and flow cytometry staining of tumor infiltrating leukocytes - Additional proteins. Related to Figure 5. Gates in each plot show the frequency of the indicated stained protein by CyTOF (left plots) or FC (right plots). Graphs display donor sample paired expression of the frequency staining positive for the indicated protein from tumor tissues by CyTOF (Cy) and FC (Fl). Significance was determined by the TOST test for equivalence. p ≤ 0.05 was considered statistically equivalent.
