# Supplementary figures and images for: Validation of CyTOF Against Flow Cytometry for Immunological Studies and Monitoring of Human Cancer Clinical Trials
Source: Front Oncol. 2019 May 17;9:415. doi: 10.3389/fonc.2019.00415 (PMC6534060; doi:10.3389/fonc.2019.00415)

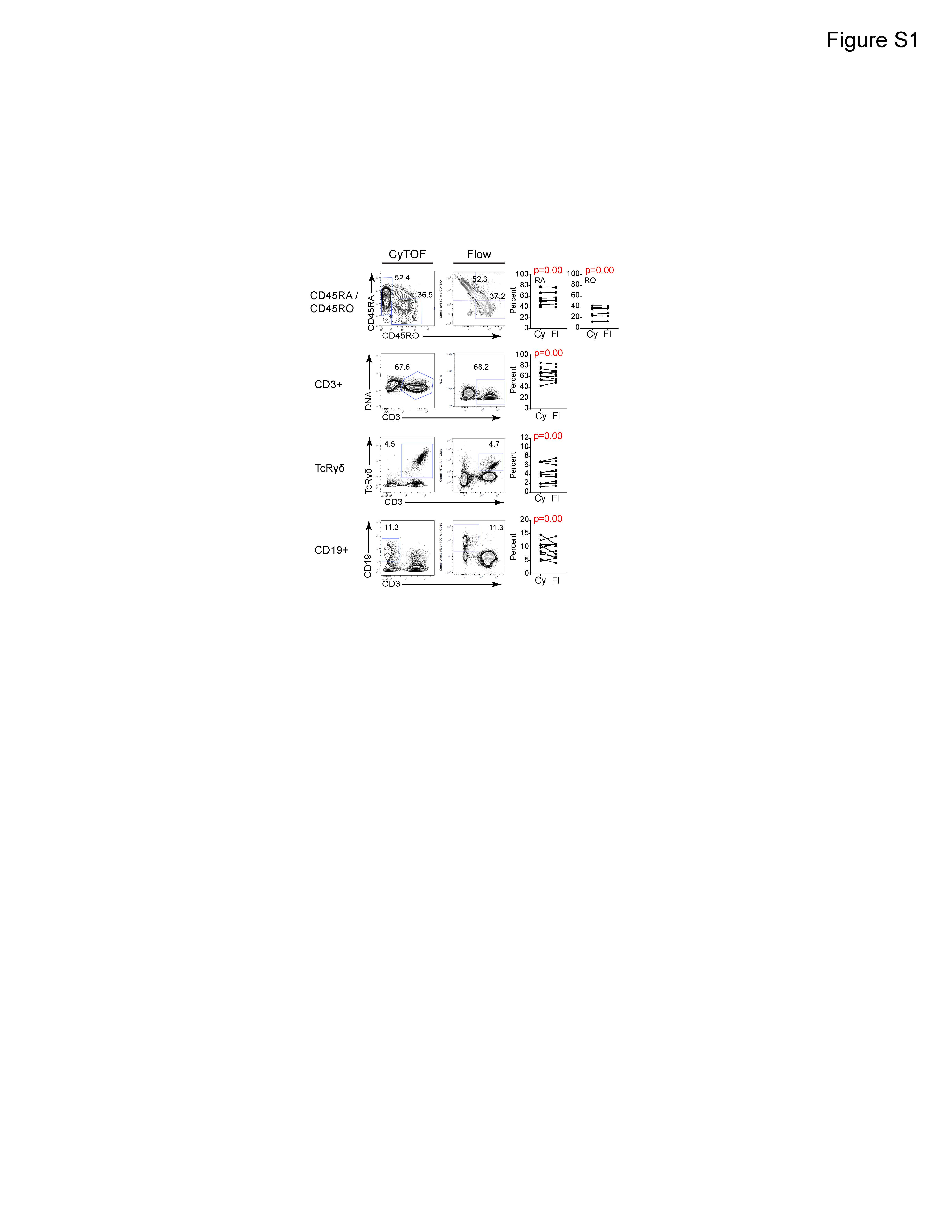

Supplement: Figure S1 — Comparison of CyTOF and flow cytometry staining of freshly isolated human PBMC- Additional proteins. Related to Figure 2. Gates in each plot show the frequency of the indicated stained protein by CyTOF (left plots) or FC (right plots). Stains were performed in the same set as shown in Figure 2. Significance was determined by the TOST test for equivalence. p ≤ 0.05 was considered statistically equivalent. [file Image_1.JPEG]

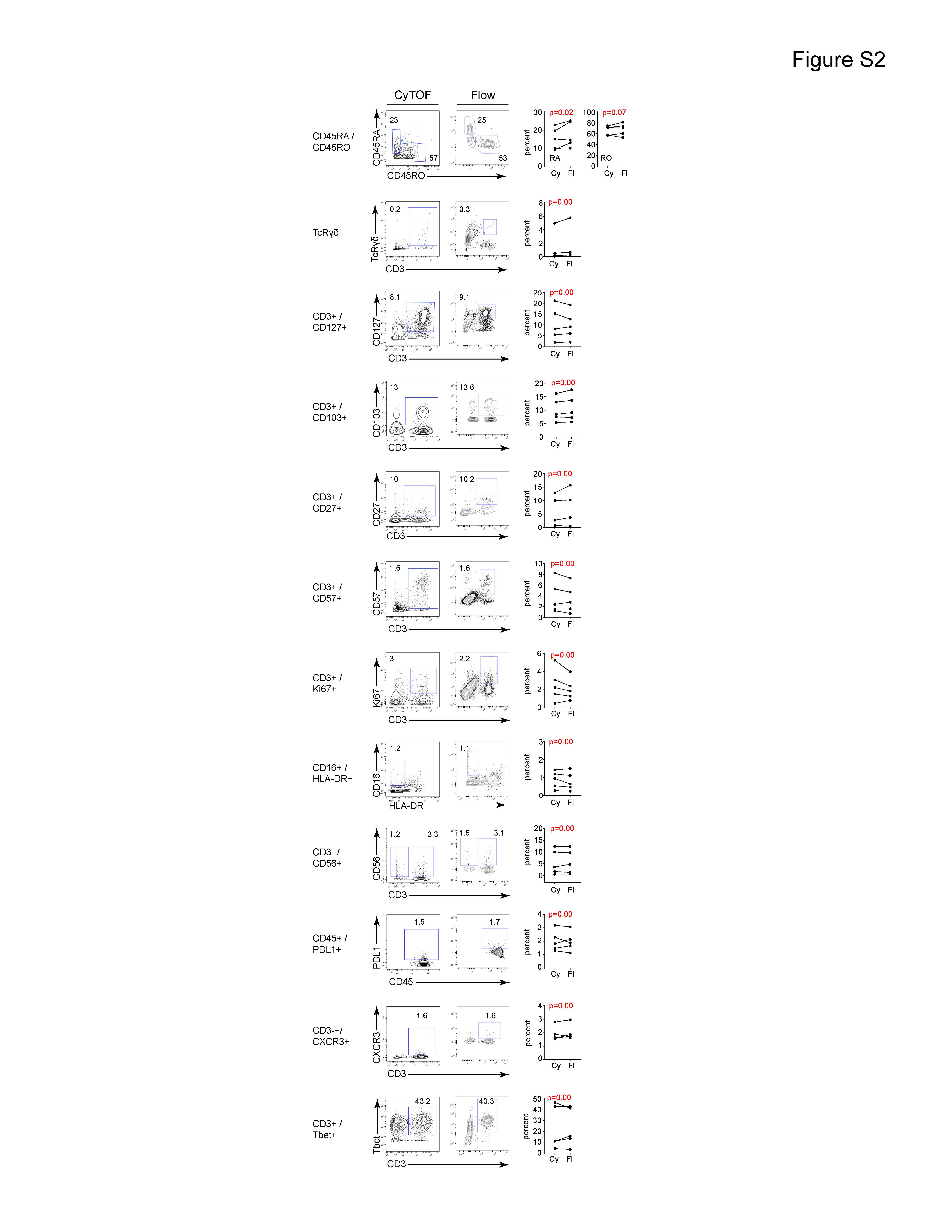

Supplement: Figure S2 — Comparison of CyTOF and flow cytometry staining of tumor infiltrating leukocytes - Additional proteins. Related to Figure 5. Gates in each plot show the frequency of the indicated stained protein by CyTOF (left plots) or FC (right plots). Graphs display donor sample paired expression of the frequency staining positive for the indicated protein from tumor tissues by CyTOF (Cy) and FC (Fl). Significance was determined by the TOST test for equivalence. p ≤ 0.05 was considered statistically equivalent. [file Image_2.JPEG]
